# 1,3-Dimethyl-2,6-diphenyl­piperidin-4-one

**DOI:** 10.1107/S1600536809003419

**Published:** 2009-01-31

**Authors:** P. Nithya, Venkatesha R. Hathwar, T. Maiyalagan, Canan Kazak, F. Nawaz Khan

**Affiliations:** a Chemistry Division, School of Science and Humanities, VIT University, Vellore 632 014, Tamil Nadu, India; b Solid State and Structural Chemistry Unit, Indian Institute of Science, Bangalore 560 012, Karnataka, India; c Ondokuz Mayıs University, Arts and Sciences Faculty, Department of Physics, 55139 Samsun, Turkey

## Abstract

In the title moleclue, C_19_H_21_NO, the 4-piperidone ring adopts a chair conformation in which the two benzene rings and the methyl group attached to C atoms all have equatorial orientations. In the crystal structure, centrosymmetric dimers are formed through weak inter­molecular C—H⋯O hydrogen bonds [the dihedral angle between the aromatic rings is 58.51 (5)°].

## Related literature

For general background, see: Badorrey *et al.* (1999 ▶); Grishina *et al.* (1994 ▶); Nalanishi *et al.* (1974 ▶); Perumal *et al.* (2001 ▶); Ponnuswamy *et al.* (2002 ▶). For a related crystal structure, see: Gayathri *et al.* (2008 ▶). For the synthetis, see: Noller & Baliah (1948 ▶). For puckering and asymmetry parameters, see: Cremer & Pople (1975 ▶); Nardelli (1983 ▶, 1995 ▶).
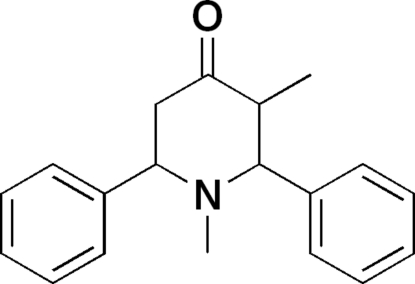


## Experimental

### 

#### Crystal data

C_19_H_21_NO
*M* *_r_* = 279.37
Triclinic, 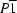

*a* = 5.9201 (2) Å
*b* = 10.9749 (3) Å
*c* = 12.8247 (3) Å
α = 80.2961 (12)°
β = 86.673 (2)°
γ = 76.4499 (11)°
*V* = 798.30 (4) Å^3^
*Z* = 2
Mo *K*α radiation
μ = 0.07 mm^−1^
*T* = 290 (2) K
0.28 × 0.21 × 0.18 mm

#### Data collection

Bruker SMART CCD area-detector diffractometer
Absorption correction: multi-scan (*SADABS*; Sheldrick, 1996 ▶) *T*_min_ = 0.943, *T*_max_ = 0.987
12316 measured reflections
3143 independent reflections
2446 reflections with *I* > 2σ(*I*)
*R*_int_ = 0.019

#### Refinement

*R*[*F* ^2^ > 2σ(*F* ^2^)] = 0.040
*wR*(*F* ^2^) = 0.116
*S* = 1.02
3143 reflections
192 parameters
H-atom parameters constrained
Δρ_max_ = 0.14 e Å^−3^
Δρ_min_ = −0.15 e Å^−3^

### 

Data collection: *SMART* (Bruker, 2004 ▶); cell refinement: *SAINT* (Bruker, 2004 ▶); data reduction: *SAINT*; program(s) used to solve structure: *SHELXTL* (Sheldrick, 2008 ▶); program(s) used to refine structure: *SHELXL97* (Sheldrick, 2008 ▶); molecular graphics: *ORTEP-3* (Farrugia, 1997 ▶) and *PLATON* (Spek, 2003 ▶); software used to prepare material for publication: *PLATON*.

## Supplementary Material

Crystal structure: contains datablocks global, I. DOI: 10.1107/S1600536809003419/lh2761sup1.cif

Structure factors: contains datablocks I. DOI: 10.1107/S1600536809003419/lh2761Isup2.hkl

Additional supplementary materials:  crystallographic information; 3D view; checkCIF report

## Figures and Tables

**Table 1:** Hydrogen-bond geometry (Å, °)

*D*—H⋯*A*	*D*—H	H⋯*A*	*D*⋯*A*	*D*—H⋯*A*
C1—H1⋯O1^i^	0.98	2.56	3.3535 (16)	139

Symmetry code: (i) 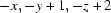.

**Fig. 1.**
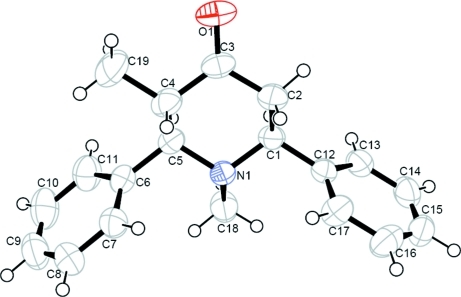
The molecular structure of (I) shown with 50% probability displacement ellipsoids.

**Fig. 2.**
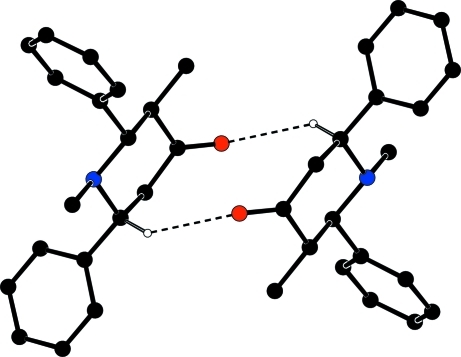
Part of the crystal structure of (I) with dashed lines indicating intermolecular C—H···O hydrogen bonds.

## supplementary crystallographic information

### Comment

The synthesis of 4-piperidones is of current interest due to their potential medical applications (Grishina *et al.*, 1994, Ponnuswamy *et al.*, 2002). 4-Piperidones have been found to exhibit blood cholesterol-lowering activities (Nalanishi *et al.*, 1974). Various piperidones and piperidine derivatives are present in numerous alkaloids (Badorrey *et al.*, 1999). Piperidones are also reported to possess analgesic, anti-inflammatory, central nervous system (*CNS*), local anaesthetic, anticancer and antimicrobial activity (Perumal *et al.*, 2001).

In the title molecule, C_19_H_20_NO (Fig. 1), the piperidine ring adopts a chair conformation (Cremer & Pople, 1975; Nardelli, 1995). The phenyl rings at positions 2 and 6 and the methyl group attached at position 3 all have equatorial orientations. In the related crystal structure of r-2,c-6-Bis(4-chlorophenyl)-t-3-isopropyl-1-nitrosopiperidin-4-one, the piperidine ring also adopts a chair conformation (Gayathri *et al.*, 2008) but the three substituents on the C atoms of the ring are in axial orientations. In the crystal structure, centrosymmetric dimers are formed through weak intermolecular C—H···O hydrogen bonds (Fig. 2).

### Experimental

The sythesis was based on a procedure in the literature (Noller & Baliah, 1948). Benzaldehyde (0.20 mol), 3-methyl-2-butanone (0.10 mol) and ammonium acetate (0.10 mol) were dissolved in 80 ml of distilled ethanol and heated over a boiling water bath, with shaking until a yellow colour developed and ultimately changed to orange. The solution was left undisturbed for 14 h. The precipitated solid was filtered and purified by recrystallization from ethanol. The piperidone intermediate was then dissolved in acetone and was alkyalted with methyliodide in the presence of potassium carbonate.

### Refinement

All H atoms in were positioned geometrically and refined using a riding model with C—H bond lenghts of 0.93, 0.97 and 0.96Å for aromatic, methylene and methyl H atoms, respectively and *U*_iso_(H) = 1.2*U*_eq_(C) or 1.5*U*_eq_(C) for methyl bound H atoms.

### Crystal data

**Table:**

C_19_H_21_NO	*Z* = 2
*M**_r_* = 279.37	*F*(000) = 300
Triclinic, *P*1	*D*_x_ = 1.162 Mg m^−^^3^
Hall symbol: -P 1	Mo *K*α radiation, λ = 0.71073 Å
*a* = 5.9201 (2) Å	Cell parameters from 873 reflections
*b* = 10.9749 (3) Å	θ = 1.9–20.8°
*c* = 12.8247 (3) Å	µ = 0.07 mm^−^^1^
α = 80.2961 (12)°	*T* = 290 K
β = 86.673 (2)°	Block, colorless
γ = 76.4499 (11)°	0.28 × 0.21 × 0.18 mm
*V* = 798.30 (4) Å^3^	

### Data collection

**Table:**

Bruker SMART CCD area-detector diffractometer	3143 independent reflections
Radiation source: fine-focus sealed tube	2446 reflections with *I* > 2σ(*I*)
graphite	*R*_int_ = 0.019
φ and ω scans	θ_max_ = 26.0°, θ_min_ = 1.6°
Absorption correction: multi-scan (*SADABS*; Sheldrick, 1996)	*h* = −6→7
*T*_min_ = 0.943, *T*_max_ = 0.987	*k* = −13→13
12316 measured reflections	*l* = −15→14

### Refinement

**Table:**

Refinement on *F*^2^	Primary atom site location: structure-invariant direct methods
Least-squares matrix: full	Secondary atom site location: difference Fourier map
*R*[*F*^2^ > 2σ(*F*^2^)] = 0.040	Hydrogen site location: inferred from neighbouring sites
*wR*(*F*^2^) = 0.116	H-atom parameters constrained
*S* = 1.02	*w* = 1/[σ^2^(*F*_o_^2^) + (0.0539*P*)^2^ + 0.1409*P*] where *P* = (*F*_o_^2^ + 2*F*_c_^2^)/3
3143 reflections	(Δ/σ)_max_ < 0.001
192 parameters	Δρ_max_ = 0.14 e Å^−^^3^
0 restraints	Δρ_min_ = −0.14 e Å^−^^3^

### Special details

**Table:**

Geometry. All e.s.d.'s (except the e.s.d. in the dihedral angle between two l.s. planes) are estimated using the full covariance matrix. The cell e.s.d.'s are taken into account individually in the estimation of e.s.d.'s in distances, angles and torsion angles; correlations between e.s.d.'s in cell parameters are only used when they are defined by crystal symmetry. An approximate (isotropic) treatment of cell e.s.d.'s is used for estimating e.s.d.'s involving l.s. planes.
Refinement. Refinement of *F*^2^ against ALL reflections. The weighted *R*-factor *wR* and goodness of fit *S* are based on *F*^2^, conventional *R*-factors *R* are based on *F*, with *F* set to zero for negative *F*^2^. The threshold expression of *F*^2^ > σ(*F*^2^) is used only for calculating *R*-factors(gt) *etc*. and is not relevant to the choice of reflections for refinement. *R*-factors based on *F*^2^ are statistically about twice as large as those based on *F*, and *R*- factors based on ALL data will be even larger.

### Fractional atomic coordinates and isotropic or equivalent isotropic displacement parameters (Å^2^)

**Table:**

	*x*	*y*	*z*	*U*_iso_*/*U*_eq_	
N1	0.17010 (18)	0.40892 (9)	0.73876 (8)	0.0419 (3)	
O1	0.25711 (17)	0.47960 (11)	1.03053 (7)	0.0615 (3)	
C1	0.1134 (2)	0.54013 (12)	0.76264 (10)	0.0419 (3)	
H1	−0.0457	0.5582	0.7916	0.050*	
C2	0.2788 (3)	0.55376 (14)	0.84570 (11)	0.0532 (4)	
H2A	0.4336	0.5475	0.8146	0.064*	
H2B	0.2276	0.6370	0.8665	0.064*	
C3	0.2882 (2)	0.45443 (14)	0.94154 (10)	0.0485 (3)	
C4	0.3367 (3)	0.32119 (14)	0.91755 (11)	0.0532 (4)	
H4	0.4923	0.3030	0.8850	0.064*	
C5	0.1607 (2)	0.31444 (12)	0.83478 (10)	0.0432 (3)	
H5	0.0040	0.3339	0.8661	0.052*	
C6	0.2066 (2)	0.18211 (12)	0.80603 (10)	0.0467 (3)	
C7	0.4060 (3)	0.13480 (14)	0.75020 (12)	0.0571 (4)	
H7	0.5153	0.1837	0.7319	0.069*	
C8	0.4446 (3)	0.01556 (15)	0.72124 (14)	0.0711 (5)	
H8	0.5799	−0.0148	0.6840	0.085*	
C9	0.2867 (4)	−0.05793 (15)	0.74677 (15)	0.0748 (5)	
H9	0.3126	−0.1374	0.7261	0.090*	
C10	0.0903 (4)	−0.01377 (16)	0.80298 (17)	0.0791 (5)	
H10	−0.0170	−0.0639	0.8214	0.095*	
C11	0.0495 (3)	0.10595 (15)	0.83308 (14)	0.0656 (4)	
H11	−0.0846	0.1348	0.8717	0.079*	
C12	0.1264 (2)	0.63589 (12)	0.66384 (10)	0.0428 (3)	
C13	−0.0613 (3)	0.73494 (13)	0.63339 (12)	0.0536 (4)	
H13	−0.1987	0.7419	0.6733	0.064*	
C14	−0.0475 (3)	0.82404 (14)	0.54407 (13)	0.0658 (4)	
H14	−0.1752	0.8902	0.5245	0.079*	
C15	0.1535 (3)	0.81493 (15)	0.48471 (13)	0.0675 (5)	
H15	0.1629	0.8751	0.4252	0.081*	
C16	0.3413 (3)	0.71672 (17)	0.51319 (13)	0.0687 (5)	
H16	0.4776	0.7101	0.4725	0.082*	
C17	0.3284 (3)	0.62776 (15)	0.60201 (12)	0.0579 (4)	
H17	0.4565	0.5616	0.6207	0.070*	
C18	0.0031 (3)	0.39902 (14)	0.66175 (12)	0.0623 (4)	
H18A	−0.1504	0.4132	0.6928	0.093*	
H18B	0.0439	0.3158	0.6422	0.093*	
H18C	0.0069	0.4616	0.5999	0.093*	
C19	0.3365 (4)	0.22361 (19)	1.01678 (14)	0.0942 (7)	
H19A	0.4369	0.2368	1.0679	0.141*	
H19B	0.3910	0.1398	0.9993	0.141*	
H19C	0.1814	0.2326	1.0458	0.141*	

### Atomic displacement parameters (Å^2^)

**Table:**

	*U*^11^	*U*^22^	*U*^33^	*U*^12^	*U*^13^	*U*^23^
N1	0.0505 (6)	0.0408 (5)	0.0341 (6)	−0.0089 (5)	−0.0065 (4)	−0.0056 (4)
O1	0.0534 (6)	0.0987 (8)	0.0390 (6)	−0.0236 (5)	0.0017 (4)	−0.0217 (5)
C1	0.0439 (7)	0.0439 (7)	0.0377 (7)	−0.0076 (5)	−0.0009 (5)	−0.0097 (5)
C2	0.0664 (9)	0.0558 (8)	0.0426 (8)	−0.0190 (7)	−0.0068 (6)	−0.0132 (6)
C3	0.0408 (7)	0.0708 (9)	0.0369 (7)	−0.0152 (6)	−0.0045 (5)	−0.0120 (6)
C4	0.0567 (8)	0.0602 (8)	0.0391 (7)	−0.0083 (7)	−0.0098 (6)	−0.0021 (6)
C5	0.0426 (7)	0.0471 (7)	0.0378 (7)	−0.0091 (5)	0.0007 (5)	−0.0032 (5)
C6	0.0483 (8)	0.0455 (7)	0.0437 (7)	−0.0102 (6)	−0.0050 (6)	0.0006 (6)
C7	0.0632 (9)	0.0483 (8)	0.0588 (9)	−0.0138 (7)	0.0082 (7)	−0.0069 (7)
C8	0.0846 (12)	0.0519 (9)	0.0729 (11)	−0.0073 (8)	0.0069 (9)	−0.0134 (8)
C9	0.0964 (14)	0.0446 (8)	0.0832 (12)	−0.0136 (9)	−0.0167 (10)	−0.0081 (8)
C10	0.0831 (13)	0.0558 (9)	0.1021 (14)	−0.0323 (9)	−0.0123 (11)	0.0036 (10)
C11	0.0572 (9)	0.0586 (9)	0.0795 (11)	−0.0193 (7)	0.0017 (8)	0.0004 (8)
C12	0.0511 (7)	0.0410 (6)	0.0388 (7)	−0.0118 (5)	−0.0038 (5)	−0.0105 (5)
C13	0.0614 (9)	0.0448 (7)	0.0522 (8)	−0.0046 (6)	−0.0024 (6)	−0.0112 (6)
C14	0.0908 (12)	0.0407 (7)	0.0607 (10)	−0.0037 (7)	−0.0145 (9)	−0.0052 (7)
C15	0.1055 (14)	0.0528 (9)	0.0486 (9)	−0.0318 (9)	−0.0065 (9)	0.0003 (7)
C16	0.0750 (11)	0.0822 (11)	0.0516 (9)	−0.0312 (9)	0.0058 (8)	−0.0018 (8)
C17	0.0536 (9)	0.0651 (9)	0.0509 (9)	−0.0106 (7)	0.0006 (6)	−0.0018 (7)
C18	0.0852 (11)	0.0505 (8)	0.0540 (9)	−0.0167 (7)	−0.0292 (8)	−0.0049 (7)
C19	0.146 (2)	0.0797 (12)	0.0527 (11)	−0.0258 (12)	−0.0310 (11)	0.0125 (9)

### Geometric parameters (Å, °)

**Table:**

N1—C18	1.4711 (16)	C9—C10	1.365 (3)
N1—C5	1.4777 (15)	C9—H9	0.9300
N1—C1	1.4800 (15)	C10—C11	1.395 (2)
O1—C3	1.2128 (15)	C10—H10	0.9300
C1—C12	1.5145 (18)	C11—H11	0.9300
C1—C2	1.5363 (18)	C12—C13	1.3820 (19)
C1—H1	0.9800	C12—C17	1.390 (2)
C2—C3	1.493 (2)	C13—C14	1.387 (2)
C2—H2A	0.9700	C13—H13	0.9300
C2—H2B	0.9700	C14—C15	1.367 (2)
C3—C4	1.503 (2)	C14—H14	0.9300
C4—C19	1.519 (2)	C15—C16	1.372 (2)
C4—C5	1.5520 (18)	C15—H15	0.9300
C4—H4	0.9800	C16—C17	1.381 (2)
C5—C6	1.5175 (18)	C16—H16	0.9300
C5—H5	0.9800	C17—H17	0.9300
C6—C11	1.383 (2)	C18—H18A	0.9600
C6—C7	1.384 (2)	C18—H18B	0.9600
C7—C8	1.384 (2)	C18—H18C	0.9600
C7—H7	0.9300	C19—H19A	0.9600
C8—C9	1.364 (3)	C19—H19B	0.9600
C8—H8	0.9300	C19—H19C	0.9600
			
C18—N1—C5	109.46 (10)	C8—C9—C10	119.37 (16)
C18—N1—C1	108.63 (10)	C8—C9—H9	120.3
C5—N1—C1	111.85 (9)	C10—C9—H9	120.3
N1—C1—C12	111.33 (10)	C9—C10—C11	120.50 (16)
N1—C1—C2	110.45 (10)	C9—C10—H10	119.7
C12—C1—C2	109.68 (10)	C11—C10—H10	119.7
N1—C1—H1	108.4	C6—C11—C10	120.59 (16)
C12—C1—H1	108.4	C6—C11—H11	119.7
C2—C1—H1	108.4	C10—C11—H11	119.7
C3—C2—C1	112.04 (11)	C13—C12—C17	118.07 (13)
C3—C2—H2A	109.2	C13—C12—C1	120.95 (12)
C1—C2—H2A	109.2	C17—C12—C1	120.97 (12)
C3—C2—H2B	109.2	C12—C13—C14	120.86 (15)
C1—C2—H2B	109.2	C12—C13—H13	119.6
H2A—C2—H2B	107.9	C14—C13—H13	119.6
O1—C3—C2	122.76 (13)	C15—C14—C13	120.18 (15)
O1—C3—C4	123.25 (13)	C15—C14—H14	119.9
C2—C3—C4	113.99 (11)	C13—C14—H14	119.9
C3—C4—C19	112.28 (13)	C14—C15—C16	119.84 (15)
C3—C4—C5	108.87 (11)	C14—C15—H15	120.1
C19—C4—C5	112.70 (13)	C16—C15—H15	120.1
C3—C4—H4	107.6	C15—C16—C17	120.21 (16)
C19—C4—H4	107.6	C15—C16—H16	119.9
C5—C4—H4	107.6	C17—C16—H16	119.9
N1—C5—C6	110.17 (10)	C16—C17—C12	120.83 (14)
N1—C5—C4	110.93 (10)	C16—C17—H17	119.6
C6—C5—C4	110.79 (10)	C12—C17—H17	119.6
N1—C5—H5	108.3	N1—C18—H18A	109.5
C6—C5—H5	108.3	N1—C18—H18B	109.5
C4—C5—H5	108.3	H18A—C18—H18B	109.5
C11—C6—C7	117.95 (13)	N1—C18—H18C	109.5
C11—C6—C5	121.26 (13)	H18A—C18—H18C	109.5
C7—C6—C5	120.78 (12)	H18B—C18—H18C	109.5
C6—C7—C8	120.81 (15)	C4—C19—H19A	109.5
C6—C7—H7	119.6	C4—C19—H19B	109.5
C8—C7—H7	119.6	H19A—C19—H19B	109.5
C9—C8—C7	120.77 (17)	C4—C19—H19C	109.5
C9—C8—H8	119.6	H19A—C19—H19C	109.5
C7—C8—H8	119.6	H19B—C19—H19C	109.5
			
C18—N1—C1—C12	−59.74 (14)	N1—C5—C6—C7	55.01 (16)
C5—N1—C1—C12	179.33 (10)	C4—C5—C6—C7	−68.12 (16)
C18—N1—C1—C2	178.16 (11)	C11—C6—C7—C8	0.9 (2)
C5—N1—C1—C2	57.22 (13)	C5—C6—C7—C8	−178.08 (14)
N1—C1—C2—C3	−52.14 (15)	C6—C7—C8—C9	0.2 (3)
C12—C1—C2—C3	−175.21 (11)	C7—C8—C9—C10	−1.1 (3)
C1—C2—C3—O1	−127.65 (13)	C8—C9—C10—C11	0.8 (3)
C1—C2—C3—C4	51.65 (16)	C7—C6—C11—C10	−1.1 (2)
O1—C3—C4—C19	1.3 (2)	C5—C6—C11—C10	177.82 (14)
C2—C3—C4—C19	−178.02 (14)	C9—C10—C11—C6	0.3 (3)
O1—C3—C4—C5	126.78 (13)	N1—C1—C12—C13	124.49 (13)
C2—C3—C4—C5	−52.51 (15)	C2—C1—C12—C13	−112.96 (14)
C18—N1—C5—C6	56.39 (14)	N1—C1—C12—C17	−56.44 (15)
C1—N1—C5—C6	176.84 (10)	C2—C1—C12—C17	66.11 (15)
C18—N1—C5—C4	179.44 (11)	C17—C12—C13—C14	−0.4 (2)
C1—N1—C5—C4	−60.11 (13)	C1—C12—C13—C14	178.66 (12)
C3—C4—C5—N1	55.96 (14)	C12—C13—C14—C15	0.0 (2)
C19—C4—C5—N1	−178.79 (13)	C13—C14—C15—C16	0.5 (2)
C3—C4—C5—C6	178.65 (11)	C14—C15—C16—C17	−0.5 (2)
C19—C4—C5—C6	−56.09 (17)	C15—C16—C17—C12	0.0 (2)
N1—C5—C6—C11	−123.92 (14)	C13—C12—C17—C16	0.5 (2)
C4—C5—C6—C11	112.95 (15)	C1—C12—C17—C16	−178.65 (13)

### Hydrogen-bond geometry (Å, °)

**Table:**

*D*—H···*A*	*D*—H	H···*A*	*D*···*A*	*D*—H···*A*
C1—H1···O1^i^	0.98	2.56	3.3535 (16)	139

Symmetry codes: (i) −*x*, −*y*+1, −*z*+2.

## References

[bb1] Badorrey, R., Cativiela, C., Diaz-de-Villegas, M. D. & Galvez, J. A. (1999). *Tetrahedron*, **55**, 7601–7612.

[bb2] Bruker (2004). *SMART* and *SAINT* Bruker AXS Inc., Madison, Wisconsin, USA.

[bb3] Cremer, D. & Pople, J. A. (1975). *J. Am. Chem. Soc.* **97**, 1354–1358.

[bb4] Farrugia, L. J. (1997). *J. Appl. Cryst.* **30**, 565.

[bb5] Gayathri, P., Thiruvalluvar, A., Manimekalai, A., Sivakumar, S. & Butcher, R. J. (2008). *Acta Cryst.* E**64**, o1973. 10.1107/S1600536808029723 PMC2959467 21201173

[bb6] Grishina, G. V., Gaidarova, E. L. & Zefirov, N. S. (1994). *Chem. Heterocycl. Compd.* **30**, 401–1426.

[bb7] Nalanishi, M., Shiraki, M., Kobayakawa, T. & Kobayashi, R. (1974). Jpn Patent 74-03987.

[bb8] Nardelli, M. (1983). *Acta Cryst.* C**39**, 1141–1142.

[bb9] Nardelli, M. (1995). *J. Appl. Cryst.* **28**, 659.

[bb10] Noller, C. & Baliah, V. (1948). *J. Am. Chem. Soc.* **70**, 3853–3855. 10.1021/ja01191a092 18121891

[bb11] Perumal, R. V., Agiraj, M. & Shanmugapandiyan, P. (2001). *Indian Drugs*, **38**, 156–159.

[bb12] Ponnuswamy, S., Venkatraj, M., Jeyaraman, R., Suresh Kumar, M., Kumaran, D. & Ponnuswamy, M. N. (2002). *Indian J. Chem. Sect. B*, **41**, 614–627.

[bb13] Sheldrick, G. M. (1996). *SADABS* University of Göttingen, Germany.

[bb14] Sheldrick, G. M. (2008). *Acta Cryst.* A**64**, 112–122. 10.1107/S0108767307043930 18156677

[bb15] Spek, A. L. (2003). *J. Appl. Cryst.* **36**, 7–13.

